# Development of Speech and Communication in Polish Children with 22q11.2 Deletion Syndrome: A Cross-Sectional Study

**DOI:** 10.3390/brainsci15010024

**Published:** 2024-12-29

**Authors:** Natalia Moćko, Marcin Rudzki, Zuzanna Miodońska, Julia Olesiak, Katarzyna Jochymczyk-Woźniak, Michał Kręcichwost

**Affiliations:** 1 Faculty of Humanities, Institute of Linguistics, University of Silesia, Sejmu Śląskiego 1, 40-001 Katowice, Poland; natalia.mocko@us.edu.pl (N.M.); julia.olesiak4@gmail.com (J.O.); 2 Faculty of Biomedical Engineering, Department of Medical Informatics and Aritificial Intelligence, Silesian University of Technology, Roosevelta 40, 41-800 Zabrze, Poland; marcin.rudzki@polsl.pl (M.R.); zuzanna.miodonska@polsl.pl (Z.M.); 3 Faculty of Biomedical Engineering, Department of Biomechatronics, Silesian University of Technology, Roosevelta 40, 41-800 Zabrze, Poland; katarzyna.jochymczyk-wozniak@polsl.pl

**Keywords:** child speech, hearing, communication, speech disfluency, 22q11 deletion syndrome, 22q11DS

## Abstract

Background/Objectives: 22q11.2 microdeletion syndrome (22q11DS) is a genetic disease caused by aberration of chromosome 22 that results in some phenotypic features and developmental disorders. This paper presents a cross-sectional study on speech and communication of Polish children with 22q11DS. Methods: Individuals affected with 22q11DS may show difficulties in functioning, including speech and hearing. Therefore, we prepared a speech development questionnaire and employed it to obtain data from parents (or legal guardians) of 54 children with 22q11DS. The questionnaire covered the following speech and communication development stages: babbling, using first words, first sentences, verbal and non-verbal communication, speech disfluencies, hearing loss, speech intelligibility, difficulties in interpersonal contact, and participation in speech therapy. The obtained answers underwent statistical analysis to verify relationships between the stages of personal development and selected dysfunctions and disorders. Results: In the study group we observed delays in achieving subsequent speech developmental stages and that hearing loss was associated with delays in producing first words. Hearing loss was reported in about a quarter of cases, but a significant proportion of children (55.56%) reported speech disfluencies, which had not been emphasized in previous works, where hearing loss is considered a common co-occurring disorder. Conclusions: Our findings suggest that this may represent a phenomenon associated with 22q11DS that warrants further investigation using standardized tests for assessing disfluencies. Additionally, we observed that speech therapists and caregivers were perceived as not fully aware of the speech development impairments caused by 22q11DS. These preliminary observations point to the need for future studies and increased awareness efforts in this area.

## 1. Introduction

22q11.2 microdeletion syndrome (22q11DS) is a genetic disease due to chromosomal aberration when a fragment of the arm of chromosome 22 is lost [1]. This results in many phenotypic features (physical and mental disorders) as well as functioning difficulties. Each patient may experience different developmental disorders, which is a characteristic feature of 22q11DS. 22q11DS is considered the most common chromosomal microdeletion disorder in humans [2,3]. According to various sources, the incidence of 22q11DS is estimated to range from 1:1000 to 1:7000 liveborn children [4,5,6,7,8,9,10,11]. However, the actual incidence of this condition may be higher than the estimates suggest. The condition may remain undiagnosed in people with mild or ambiguous symptoms, or it may be misdiagnosed as another disorder with similar features [1].

The development of children with 22q11DS is often delayed on many levels. Delayed speech development may be associated with some or all aspects of speech (phonetic, grammatical, lexical, or expressive) [12]. Some language skills of children with 22q11DS, especially verbal memory, reading, and spelling, are better than skills at the level of non-verbal processing, e.g., mathematical skills, abstract thinking, visual memory, visuospatial processing [13,14]. Some studies found that children with 22q11DS showed lower executive functioning abilities with no relation to the overall intelligence quotient (IQ) [15,16,17]. Due to the high susceptibility of people with 22q11DS to mental diseases (affecting 20% of children and 25% of adults [14]) as well as behavioral disorders (affecting 80–100% of people [18]), compensating for the delays may be challenging to the social environment.

The features of 22q11DS can be diagnosed during fetal life, especially when microdeletion was previously diagnosed in the family [1]. Diagnosis at birth is usually established when newborns present features typical of 22q11DS, such as heart defects, facial dysmorphia, cleft palate, or hypocalcemia. With less noticeable defects, diagnosis is made in older children or adolescents. Some people are diagnosed with 22q11DS in their adulthood when their offspring is diagnosed. They admit that the diagnosis of microdeletion explains their former difficulties in learning and social functioning at school [10,11]. Patients show significant variability in their clinical picture [19], which may delay the diagnostic process.

The Polish literature on 22q11DS is mainly focused on aspects of medical and therapeutic management resulting from typical congenital disabilities mostly related to the defects of the heart, respiratory, and immune systems, as well as cleft palate disorders [18,20,21,22]. Indications for speech and pedagogical therapy are given based on case studies. Such research had never been conducted on Polish cohorts [23,24].

Studies related to speech in children with 22q11DS found delays in acquiring language and articulation skills [25,26,27] as well as communication and social skills [28,29,30]. The delays can be associated with up to 95% of the 22q11DS population [12], and they are confirmed in various languages [31,32]. Some authors reported abnormalities in language sub-systems, including grammar, vocabulary, and language pragmatism [33], which may be connected with the role that the genes affected by 22q11DS played in language development [2]. Cultural differences and their impact on language skills in children with 22q11DS were also reported [34].

Persistent delay in speech development results in incorrect functioning in social and emotional life and may cause behavioral disorders [35,36,37]. For this reason, speech therapy assistance is beneficial to people with 22q11DS. However, due to time constraints, therapeutic activities are sometimes limited only to the most essential defects diagnosed in the child [38,39,40]. Therefore, some researchers discuss the need for increasing speech therapy interventions [41,42].

Previous research on Polish parents of children with 22q11DS indicated that specialists’ knowledge, including family doctors, physiotherapists, or psychologists, about the syndrome was insufficient [43]. The primary source of knowledge for parents is the Internet and associations [44,45]. This is also of concern because parents can experience anxiety, stress, and even mental disorders resulting from the insufficient knowledge of medical specialists [46]. The knowledge of speech therapists was not considered in the studies.

We described the population of Polish children with 22q11DS and their parents’ views on the effectiveness of therapeutic support. Early intervention, particularly individualized speech therapy, is crucial to prevent or reduce the impact of disability. Understanding language difficulties and the assistance offered to children with 22q11DS are essential for standardizing speech therapy procedures and improving healthcare and educational systems.

This study aimed to provide information on speech and communication development in Polish children with 22q11DS from their parents’ perspective. We constructed a speech development questionnaire and employed it to obtain the data from parents or legal guardians of children with 22q11DS in order to determine the associations between language development and various disfunctions diagnosed in children. We considered several types of dysfunctions:

- Speech development delays—studies on Polish children [47,48,49] indicated delays in achieving speech development milestones (babbling, using first words, and first sentences). Our aim was to verify whether there were any specific relations in those delays and to what extent the difficulties started with the babbling stage in the group of children with 22q11DS. Another goal was to check whether children with 22q11DS achieved a similar level of speech development as children without 22q11DS, as assessed by the parents/guardians;
- Signal diversification—we decided to check whether a child diversified their communication signals towards other people [50,51]; in other words, whether the parents/guardians noted socially effective strategies of their children. The communication signals can be non-verbal for younger children and articulative for older ones. The signals a child uses may be clear to the members of the closest social environment (e.g., parents) but not to people who have rare contact with the child. We wanted to check if parents/guardians recognized the difference in their understanding of the child’s signals compared to other individuals. However, no speech or auditory tests were performed. Therefore, the actual state may be different than the one reported in the questionnaire;
- Hearing loss and speech disfluencies—as reported in the literature [13,14,20,52], hearing loss seems to be a vital source of information on the functioning of individuals with 22q11DS. Currently, after hearing loss was found, a check for speech disfluencies was also performed (e.g., stuttering [53,54]). We wanted to gain insight on various disfluencies that could occur in children with 22q11DS. We selected the features of speech disfluencies that were indicated in the literature and could be observed by parents [55,56]. Confirmation of stuttering in children with 22q11DS would necessitate the presence of a balbutology expert in the team conducting the therapy for the child.

We compared the collected material with the literature on 22q11DS and developmental norms for the general population. In the case of Polish children with 22q11DS, issues related to speech and communication development as well as speech therapy for pediatric patients had not been studied as of yet in a population context. Based on the data and future research, we aimed to develop a comprehensive profile of the language and communication development of Polish children with 22q11DS.

## 2. Materials and Methods

### 2.1. Preparation and Data Collection

We developed our own questionnaire based on the literature [2,7,14,57,58]. The questionnaire was intended to be completed by an adult who had the most relevant information about the development of the child. The questions were related to the age of the child, time at diagnosis of 22q11DS, and disorders such as speech disfluencies, hearing loss, orofacial dysfunctions, abnormal muscle tone, and other aspects that could have an impact on the diagnosis and speech therapy. Additionally, the questionnaire included questions associated with communication features, such as the preferred way of communication, intelligibility of the messages, diversification of signals, and the ability to establish contact with another person. The questionnaire consists of 18 closed questions, 4 questions in which there is a possibility to add an answer, 10 short open questions, and 4 open questions. Next, the survey questions were consulted on with specialists (geneticist, psychiatrists, and psychologists). Finally, the questionnaire was approved by the 22q11 Poland Association. The English version of the questionnaire is attached in Appendix A.

A survey method was employed to obtain information from parents or guardians of children diagnosed with 22q11DS using the FISH19 test. Surveys in electronic form (Google Forms document) were sent by the management of the 22q11 Poland Association to parents/guardians of children diagnosed with 22q11DS (members of the Association or the Support Group for parents). The questionnaire was sent by e-mail with introductory information and contact details in case of questions or doubts. Neither the members of the Association nor the respondents indicated any inaccuracies or submitted additional comments to the questionnaire. The intention to make the questionnaire available through the Association rather than a public forum was to make it available only to parents/guardians of children with 22q11DS.

Between February and April 2023, 54 respondents provided answers to the questionnaire. They concerned children of various ages (0–18 years) and at different stages of speech development. Completing and returning the questionnaire was tantamount to giving consent to participate in this study (no personal data were collected). All data were consistent. The same child was not described twice (e.g., separately by the mother and father). We divided the research material into five age groups—intervals in which individual language skills are formed based on the Polish literature, which made it possible to determine whether a child in a given group acquired skills typical of that age interval. The adopted age division was related to the ranges for acquiring language and communication skills, as indicated in the Polish literature [47,48,49]:

(1) “infants”—under one year of age (2 participants);
(2) “1;0–1;11”—children from 1 up to 2 years of age (5 participants);
(3) “2;0–2;11”—children over 2 up to 3 years of age (7 participants);
(4) “3;0–6;11”—children over 3 up to 7 years of age (20 participants);
(5) “7;0 and over”—over seven years of age (20 participants).

In some cases, narrower sub-groups were made, and then the group was labeled as “year;0–year;11” as the starting and ending age. Similarly, broader age groups were also formed where required for the purpose of statistical analysis:

- Up to 3 years of age, covering skills such as a pre-linguistic phase to mastering words and simple sentences;
- From 3 to 7 years of age: development of language skills to produce longer, more complex utterances;
- Over seven years of age: mastering skills of descriptive and dialogical speech, as well as reading and writing skills.

### 2.2. Statistical Analysis

To investigate the tendencies in the data, we performed several statistical analyses. We analyzed age differences between groups showing different levels of speech or communication competences. In these cases, we applied the Shapiro–Wilk test to assess whether the age data in groups followed a normal distribution. Additionally, the Levene and Brown–Forsythe tests were used to check for homogeneity of variances. As the age-related data did not meet the assumptions of normal distribution or equal variance, we applied non-parametric statistical methods.

The Mann–Whitney–Wilcoxon test was used for comparisons between two groups, while the Kruskal–Wallis test was used for comparisons across multiple groups. Post hoc analyses were conducted using the Dunn’s test with Bonferroni correction to control for the risk of type I errors in multiple comparisons following significant Kruskal–Wallis results.

Additionally, cross-tabulations and the chi-square test were employed to explore relationships between categorical variables (e.g., the presence of speech disfluencies and hearing loss). In these cases, the grouping was based on different age categories of children with 22q11DS, reflecting distinct stages of speech and language development. To evaluate correlations between continuous variables, such as the age at which speech development milestones were reached, Kendall’s correlation coefficient (tau-b) was calculated. This method was chosen to find the relationships in ordinal data.

Conventional significance levels were adopted, with *p* values below 0.05 (*), 0.01 (**), and 0.001 (***) considered statistically significant. All analyses were conducted using the JASP computing environment (version 0.17.3) [59].

## 3. Results

### 3.1. Age Distribution of the Participants

**Figure 1:**
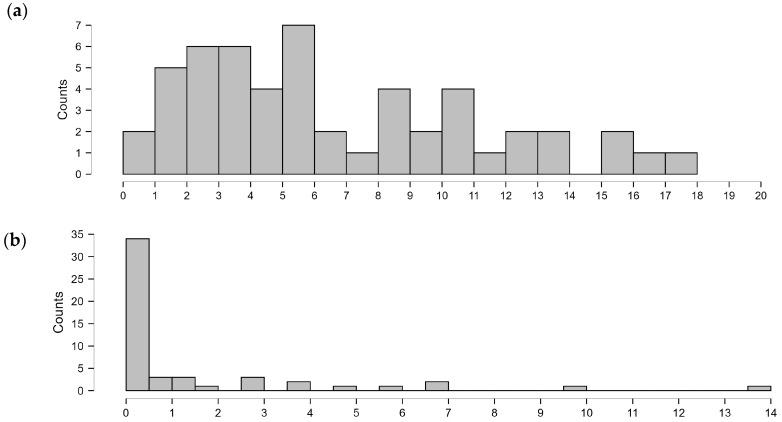
(**a**) Age (years) of the participants; (**b**) age (years) at diagnosis of 22q11DS.

Most of the subjects were within the age range in which they should have fully developed speech and communication skills in the expected components of the language system, as indicated by development standards. Figure 1a shows the age distribution of all the children participating in this study. Respondents also indicated the age at diagnosis of 22q11DS in their child (the FISH19 test) (Figure 1b). Eight people (14.81%) mentioned the circumstances of prenatal diagnosis. Detection of the syndrome before age 1;0 (years; months) was the most common, as this answer was given by 50% of respondents. On the other hand, a late diagnosis of 22q11DS is quite rare and was confirmed in three people (5.56%).

**Figure 2:**
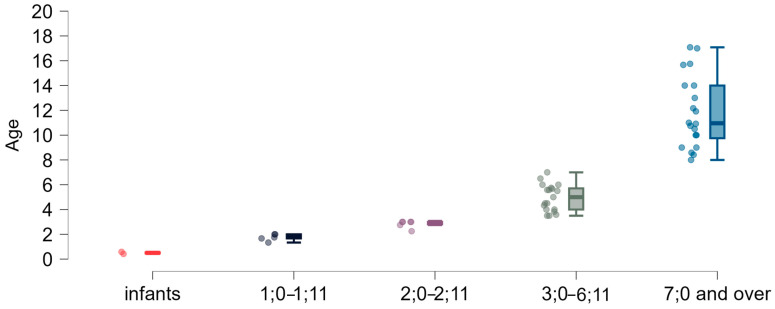
Age (years) of the study group: dispersion within individual age groups.

As shown in Figure 2, age distributions within the age sub-groups did not meet the assumptions of a normal distribution or equal variance.

### 3.2. Speech Development in Children with 22q11DS—Comparison with Developmental Norms

**Figure 3:**
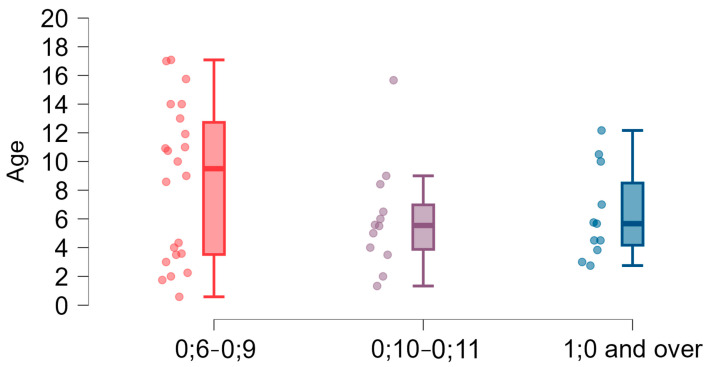
Time of babbling onset (year; month age groups) and age dispersion (years).

To determine whether babbling occurred at the right time in children with 22q11DS, the respondents were asked about the age at which children started to babble. Of 52 answers (two respondents did not provide any answer), 22 (42.30%) indicated the adequate time of babbling onset between the 6th and 9th months of age. One child was not classified into any group due to age (<1 year of age) and no babbling. In 12 children (23.07%), babbling started between the 10th and 12th month of age, which is referred to as the diverse babbling stage [60]. In 11 respondents (21.15%), babbling was delayed and started after the first year of life. A group of six children (11.53%) had not achieved the babbling stage despite being over one year of age. Respondents from the last two groups (17 people, 32.08% in total) already showed delays or difficulties in the development of speech in the period of babbling. The answers are given in Figure 3 and Table 1.

**Figure 4:**
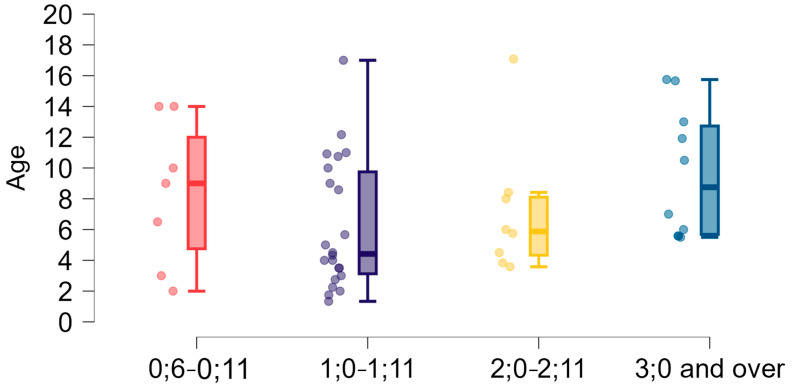
Time of the appearance of the first words (year; month age groups) and age dispersion (years).

Data on the appearance of the first words in the study group are given in Figure 4 and Table 2. Twenty-four people (44.4%) produced words at that age, and 12.96% (*n* = 7) produced words even before 12 months. However, 33.3% (*n* = 18) began this stage after the age of two, including 18.5% (*n* = 10) after three years of age, of whom five respondents pointed to the 5th or 6th year of age, when typically developing children should use free sentences.

**Figure 5:**
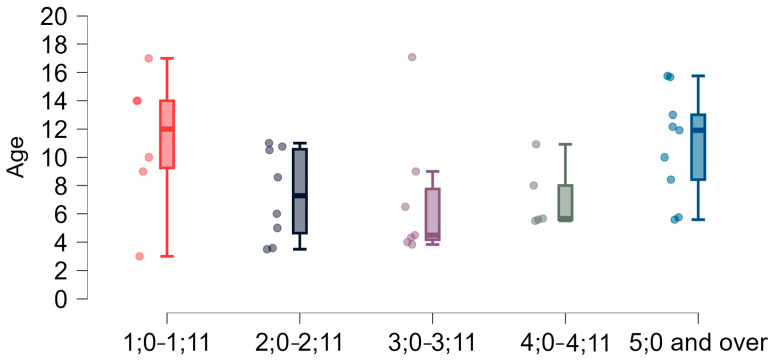
Time of the appearance of the first sentences (year; month age groups) and age dispersion (years).

The answers related to the appearance of the first sentences obtained for the population are given in Figure 5 and Table 3. This period did not apply to seven children (13.0%) because the children were at the age in which they were not able to create their first sentences. According to parents/guardians, 15 children (27.78%) formed their first sentences between 2 and 3 years of age, including 6 children (11.1%) who had mastered the skill by the time they were 2;0. This study showed that 21 children started using sentences after age 3;0 (38.89%), including 9 children at an age above five years. Nearly a fifth (*n* = 11) of the respondents did not master the ability to use sentences. As in the case of previous periods of speech development, the appearance of first sentences appeared to be delayed in the study population.

### 3.3. Correlations Between Delays in Subsequent Stages of Speech Development in Children with 22q11DS

We examined the relationships between the onset of babbling, first words, and sentences. Participants who did not reach the analyzed stage of speech development were excluded from this analysis. Table 4 shows the results of the correlation analysis.

- Babbling vs. first words (*n* = 44)—the value of the correlation coefficient (tau-b 0.368) was statistically significant (*p* < 0.001). There was a moderate positive correlation between the age of babbling onset and the age at which the first words appeared—the later the babbling occurred, the later the first words appeared;
- Babbling vs. first sentences (*n* = 33)—the value of the correlation coefficient (tau-b 0.357) was statistically significant (*p* < 0.01). The results showed a moderate positive correlation between the age of babbling and the age of first sentences. As before, delayed babbling was associated with delayed occurrence of the first sentences;
- First words vs. first sentences (*n* = 35)—the value of the correlation coefficient (tau-b 0.593) was statistically significant (*p* < 0.001). The results showed a strong positive correlation between the age at which the first words and first sentences appeared. Children who started using their first words early tended to construct complete sentences more quickly.

The relationships indicated that delays at each earlier stage of speech development prolonged the acquisition of the next milestone of language development.

### 3.4. Communication Strategies in Children with 22q11DS

**Figure 6:**
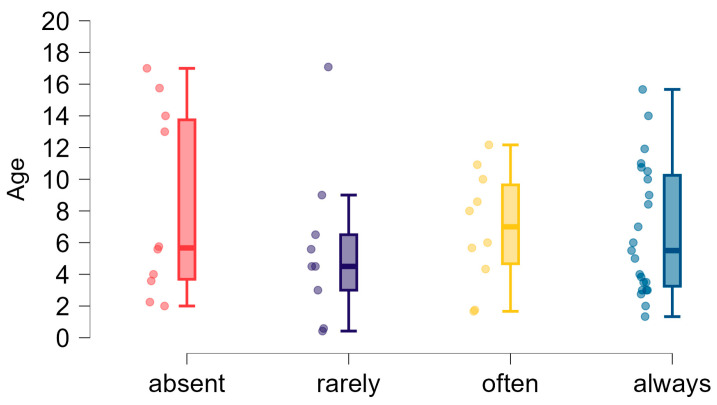
Ability to diversify verbal and non-verbal signals in children and age dispersion (years).

We asked respondents about their child’s ability to diversify signals—non-verbal and verbal indications of the child’s needs and emotional states that were understandable to their environment. As indicated in the introduction, diversification of signals occurs when a child indicates, e.g., hunger differently than any other need in a way that is comprehensive to others. Respondents assessed the frequency of their child’s ability to diversify signals on a four-point scale (always, often, rarely, or absent). The distribution of individual responses is given in Figure 6.

Almost half of the respondents (46.3%, *n* = 25) chose the answer ‘always’. Therefore, the child’s communication strategy was effective from the adult’s point of view. About a third of the respondents (*n* = 19) indicated abnormalities in social communication, not diversifying signals at all (10 people of different ages, not only infants), or doing it rarely (*n* = 9).

To verify whether there is a relationship between the age of the subjects and their ability to diversify communication signals, we compared the age of participants in groups with different frequencies of signal diversification ability (always/often/rarely/absent). The data describing the age of the respondents in the groups were not normally distributed (*p* = 0.003). Therefore, we compared the median age between groups using the Kruskal–Wallis test (*p* = 0.744). The results showed no statistically significant differences in the age of children with differing abilities to differentiate communication signals.

To determine the prevailing ways of communicating with the environment in the study population, we asked guardians about how children usually communicated. The analysis of cross-tables (Table 5) showed that children from 2 to 3 years of age preferred communication using gestures (3/7) and single words (3/7). In the age group of children from 3 to 7 years of age, single words were still a common way of communicating (20% of the group, i.e., four participants). However, the number of children who started using sentences also increased (30% of the group, i.e., six participants). In the age group over 7 years, all children (*n* = 20) preferred communication in the form of longer statements. The chi-square test (35.247, *df* = 6) confirmed statistically significant relationships between the complexity of communication methods and the age groups of children (*p* < 0.001). These results showed that preferred forms of communication changed as the child developed language skills.

### 3.5. Speech Disfluencies in Children with 22q11DS

**Figure 7:**
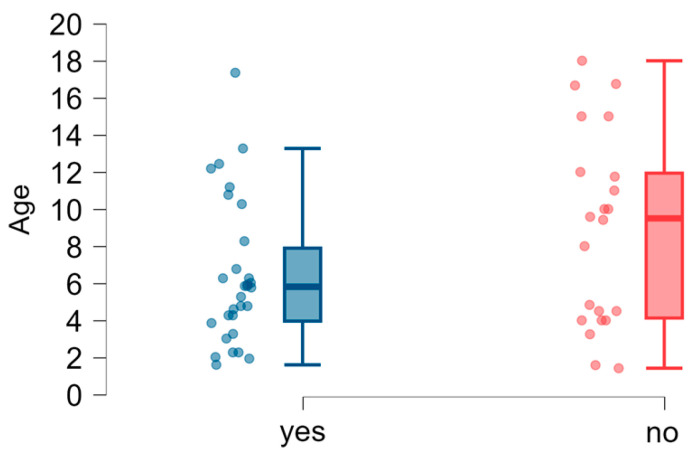
Speech disfluencies: age dispersion (years) in the groups of children with and without disfluencies.

The occurrence of disfluencies in the speech of children in the study group was often indicated by respondents (30 respondents, 55.56%, Figure 7). We conducted an analysis to examine whether the age of onset of various stages of speech development, such as babbling, first words, and first sentences, had a significant relationship with the occurrence of speech disfluencies in the study group. For this purpose, we divided the subjects into a group with speech disfluencies and a group without disfluencies. Data on the subjects’ age at milestones in the groups were not normally distributed (Shapiro–Wilk test: *p* < 0.05). Therefore, the Mann–Whitney–Wilcoxon test was used to assess the differences. The median age of children in both groups was not significantly different, which shows that the groups were homogenous. The results (Table 6) showed no significant differences in median values between the study groups in the age of babbling onset, producing first words, and first sentences. The analysis showed that the age of reaching milestones related to the child’s speech development, such as babbling, first words, and first sentences, was not correlated with the presence of speech disfluencies.

**Figure 8:**
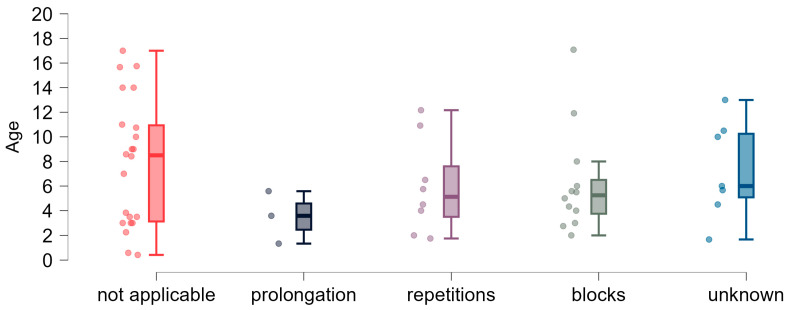
Types of observed disfluencies and age dispersion (years).

To better characterize speech disfluencies, we asked respondents about the dominant type of disfluencies (Figure 8). Of thirty respondents who emphasized the presence of disfluencies in a child’s speech, most indicated that disfluencies mainly manifested in the form of blocks (*n* = 12, 40%) or repetitions (*n* = 8, 26.7%). Three respondents indicated prolongation (which is probably more challenging to notice than blocks or repetitions). Nearly 25% of respondents (*n* = 7) were aware of the lack of fluency in children’s communication but could not determine the type of the disfluencies.

### 3.6. Hearing Loss in Children with 22q11DS

**Figure 9:**
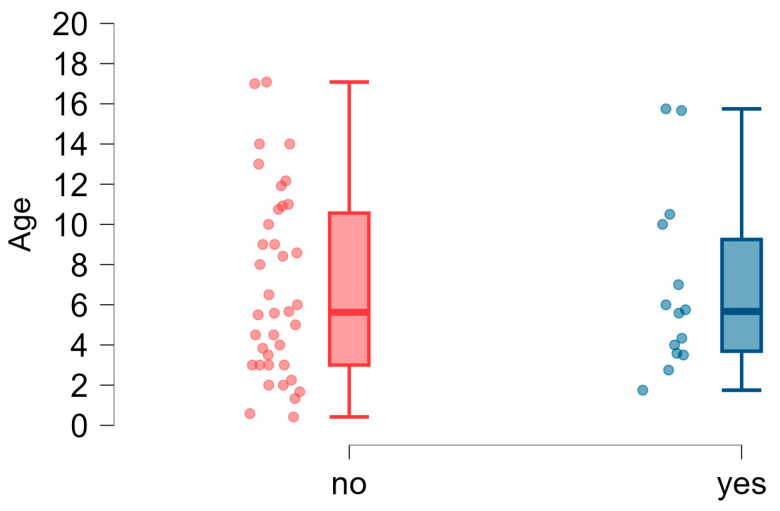
Reported hearing loss in the study group and age dispersion (years).

In our study on Polish children with 22q11DS, hearing loss was reported in fewer individuals (Figure 9) than disfluencies (Figure 7)—the diagnosis of hearing loss was confirmed by 26% of respondents (*n* = 14). Therefore, hearing loss, indicated in the literature as a characteristic co-occurring disorder of the syndrome, was less common in this study group than disfluencies, which had not been reported by other researchers before.

We conducted statistical analysis to examine whether the age of occurrence of subsequent speech development milestones, such as babbling, first words, and first sentences, had a significant relationship with the occurrence of hearing loss in the study group. We compared the groups with hearing loss and normal hearing. Data on age at milestones were not normally distributed (Shapiro–Wilk; *p* < 0.05). Therefore, we used the Mann–Whitney–Wilcoxon test to assess the differences. The results (Table 7) showed no significant differences in the median values of age in the context of babbling and first sentences related to hearing between the groups. However, significant differences were found between the groups in terms of the age of using the first words (*p* = 0.011). Therefore, hearing loss in the study population can be associated with later vocabulary development. However, it should be emphasized that parents/guardians were not asked when hearing loss first occurred but whether it had ever been diagnosed.

In our dataset, orofacial dysfunction occurred in 71.4% of the respondents diagnosed with hearing loss (*n* = 10). Table 8 shows the results of the cross-analysis between the two variables, i.e., the occurrence of hearing loss and orofacial dysfunction. The analysis using the chi-square test did not indicate a statistically significant relationship between the occurrence of hearing loss and orofacial dysfunction in the study population of 54 people (*p* = 0.450). However, it should be noted that the group characterized by hearing loss without orofacial dysfunction was very small (*n* = 4).

### 3.7. Communication Skills in Children with 22q11DS—Intelligibility and Speech Development

Considering the previous observations, i.e., developmental abnormalities, delays in the development of speech skills, and disorders affecting the intelligibility of messages, we decided to check whether children with 22q11DS were understood by their surroundings. For this purpose, parents and guardians were asked who understood the child’s speech (Table 9). Children under two years of age were not included in this analysis since during the first two years of life, the most frequent recipients of a child’s messages are parents/guardians only. Children over two years of age usually have more contact with a broader audience due to attending a nursery or kindergarten.

Based on the data, no statistically significant relationship was found between age groups and the child’s ability to be understood while speaking (chi-square test: 2.461, *p* = 0.652). The child’s speech was fully understandable to the environment in the case of 27.66% of children from the 4–7 age group (*n* = 13) and only 21.28% of the children over seven years of age (*n* = 10). Considering the entire study group, the speech of children with 22q11DS was understood by only one person or several listeners in the case of as many as 44.861% of the respondents (*n* = 21). Therefore, considering all age groups, almost half of the children were not sufficiently understood in their community.

### 3.8. Communication Skills in Children with 22q11DS—Difficulties in Interpersonal Contacts

We investigated the difficulties in interpersonal contact experienced by the participants. Table 10 shows the frequency of such difficulties in different age groups among the respondents. We assumed that the terms ‘never’ and ‘rarely’ were synonyms of the occurrence of difficulties (very low frequency). Infants (under one year of age) and children aged 1;0–2;11 years showed no difficulties in interpersonal contact. For children aged 3;0–3;11, the difficulties were found in three people (42.86% of this group). For children aged 4;0–6;11, eight people (40% of this group) showed difficulties. For children over the age of seven, 11 people (55% of the age group) had problems in contact with others.

We expected that difficulties in interpersonal contact would increase with the age of the respondents. Non-parametric Kendall correlation analysis was employed to detect potential correlations. The results are given in Table 11. The correlation coefficient value (tau-b: 0.317 **) was statistically significant (*p* = 0.003), which suggests a moderate positive correlation between the age of the respondents and the frequency of difficulties in terms of contact with other people.

### 3.9. Speech Therapy for Children with 22q11DS

Respondents were asked whether the child attended speech therapy, how long it lasted, and when it started. When asked whether the respondent attended speech therapy, 94.44% of respondents (51 parents/guardians) gave a positive answer (Table 12). The age of three children not attending speech therapy ranged from 1;0 to 3;11 (years; months), and all children over the age of 4 years participated in speech therapy. Therefore, we concluded that various disorders indicated the need for therapy in almost 100% of the population of children with 22q11DS over a certain age (Table 13).

**Figure 10:**
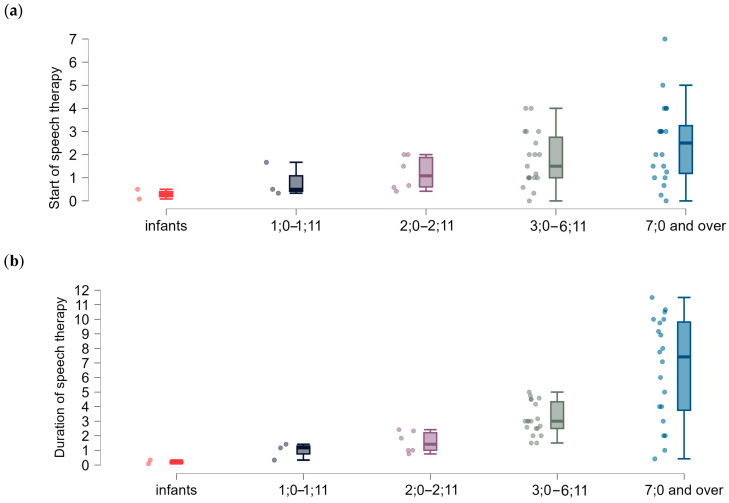
The age (years) of participants: (**a**) when starting speech therapy; (**b**) the duration of speech therapy (years).

Figure 10 shows the onset of speech therapy (a) and the duration of therapy (b) in the age groups. The mean time of therapy onset was 22 months of life (±17). The mean therapy duration was 47 months (±38). Speech therapy was completed in 15 respondents (29.41%). The remaining 36 people (70.58%) still attended speech therapy. The mean time of therapy of almost 4 years together with the information on therapy continuation by almost 70% of the participants indicated that therapy for children and adolescents with 22q11DS was prolonged.

The results of the Kendall correlation analysis between the patients’ age and the duration of speech therapy are given in Table 13. The analysis of the results showed a statistically significant positive relationship between the two variables. The values of correlation coefficients (Kendall’s tau: 0.540, *p* < 0.001) showed a tendency to increase the duration of speech therapy as the age of the patients increased.

**Figure 11:**
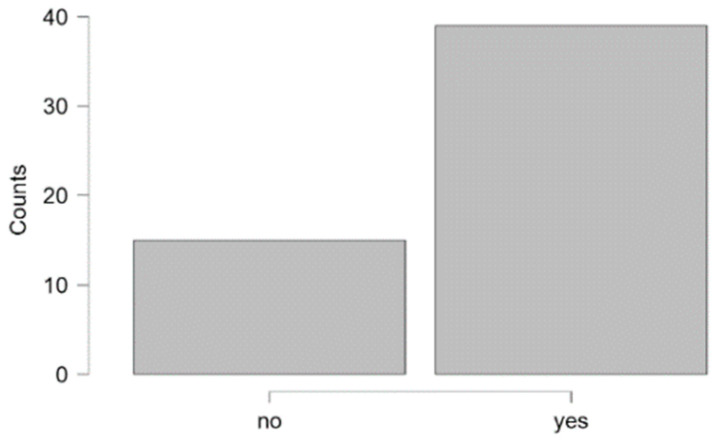
The respondents’ opinions on the question whether speech therapists had sufficient knowledge of 22q11DS.

Parents or guardians were asked whether speech therapists had sufficient knowledge of the syndrome (Figure 11). According to the majority of respondents (*n* = 43, 79.63%), speech therapists did not have sufficient knowledge about 22q11DS (disagree/strongly disagree). Only 11 people (20.37%) indicated otherwise (agree/strongly agree).

## 4. Discussion

### 4.1. Key Findings for Polish Children with 22q11DS

According to the literature, typical symptoms of 22q11DS include delayed speech development, hearing loss, orofacial dysfunction, and impaired muscle tone [2,3,12,18,24,61,62,63,64,65]. These factors are essential from the perspective of the diagnosis of and therapy for speech. However, information in this field has not yet been examined in detail in the population of Polish children.

Diagnosis of microdeletions, particularly 22q11.2, is often made before a child reaches one to three years of age. When certain abnormalities are observed in the form of developmental anomalies and dysmorphic features, their cause is diagnosed and may be associated with 22q11DS [12,21,43,63]. Children diagnosed in the first year of life often receive help due to abnormalities in the orofacial area and impaired sound reception (cleft, hearing loss). In our study, 50% of the population was diagnosed before the age of one.

Delays in achieving the stage of babbling and the subsequent developmental stages [47] were observed in our study. Babbling is part of the melody period and should occur from about six months of age [47,49]. So far, attention has been paid to the lack or limited babbling of children with 22q11DS [12,61,66]. However, such research had not been conducted on Polish cohorts. A significant proportion (32.08%) of our study group showed delays or difficulties in reaching the babbling stage. We also found that many children in the study group started speaking their first words and forming sentences later than typically expected. While 44.4% produced first words around the expected age (1–2 years), 33.3% started after the age of two, with some not forming sentences until after five years of age. This study showed that delays in early speech development milestones were significantly correlated with further achievements in language skills, which indicates that the timing of babbling, first words, and first sentences was interconnected.

This study found a significant relationship between age and the complexity of communication methods. In the population of children aged ‘7;0 and above’, parents considered their children’s speech to be developed to the level typical of school-age peers. It is consistent with the profile of a child with 22q11DS, which suggests that linguistic development is relatively well achieved compared to other developmental aspects [14].

We found a moderate positive correlation between age and the frequency of difficulties in interpersonal contacts in children with 22q11DS, which indicates that as children grow older, they tend to experience more challenges in social interactions. Specifically, difficulties were observed in 42.86% of children aged 3 years and in 55% of those over 7 years of age, which suggests that social communication issues become more prevalent with age in this population. Not being understood by the environment persisting over time may be difficult for children, which may make them reluctant to communicate [28,29,67]. Our research showed that most of the study group could experience frustration because they were not fully understood by those around them (25 people, 46.3%). This lack of understanding of the child’s messages is probably due to delays in speech development and the use of age-inappropriate communication methods, e.g., gestures, single words, or even single sounds (37 people in the study population, 68.5% of the respondents). Unintelligible speech may also be a reason, as the lack of clarity may result from cleft palate, articulation, or rhythm disorders [25,29]. Other causes include a decline in executive and linguistic functions [15,16,17,33,68].

A significant portion (55.56%) of respondents reported the presence of speech disfluencies in children with 22q11DS. The most prevalent types of disfluencies included blocks and repetitions. We found no significant differences in the age of reaching key speech development milestones (babbling, first words, and first sentences) between children with and without speech disfluencies, which suggests that the presence of disfluencies is not directly related to the timing of speech development milestones. Previous studies on 22q11DS did not include information on speech disfluency as a feature typically found in children with the deletion syndrome. Our findings show the need to expand knowledge on this phenomenon. The data can, therefore, be a starting point for “updating” the image of an individual with 22q11DS.

Hearing defects in children significantly affect speech development [69,70,71,72] and may cause delays and limit language experience [48,69,70,71]. Their presence in the case of 22q11DS is stressed in the literature [3,18,61]. Our study found that hearing loss was associated with a delay in the age of producing first words, which indicates that children with hearing loss have delayed vocabulary development. However, no significant relationships were found between hearing loss and the age of babbling or producing first sentences. In the study population, hearing loss was reported in only 26% of respondents, which is significantly lower than the prevalence of speech disfluencies (55.56%). This finding is not in line with the literature data that stresses hearing loss as a common co-occurring disorder in 22q11DS, which suggests that speech disfluencies may be more prominent in this specific population.

Speech therapy is often a necessary form of supporting the development of children with 22q11DS [2,29,33]. In our study, nearly all children over the age of 4 years (94.44%) were involved in speech therapy, which stresses significant speech and communication challenges associated with the syndrome. The mean age for starting therapy was around 22 months, with a mean duration of 47 months, which indicates that many children require long-term speech intervention due to persistent communication difficulties. Children with 22q11DS attend speech therapy due to various communication difficulties, developmental disharmony, or functional disorders, and not due to the nature of the syndrome. However, the knowledge of speech therapists of the syndrome was assessed rather negatively by the parents/guardians of Polish children. According to nearly 80% of respondents (*n* = 43), speech therapists did not have adequate information about the syndrome. The lack of common knowledge on 22q11DS may lead to overlooking the specificity of the syndrome when planning therapeutic activities. This concern underscores the need for increased education and awareness among speech therapy professionals regarding the specific needs and characteristics of children with this genetic syndrome.

### 4.2. Study Limitations

While our study provides valuable insights into the speech and communication development of Polish children with 22q11DS, several limitations must be considered.

First, our study relied on parent-reported data collected through an electronic survey rather than direct clinical evaluation by speech–language pathologists or other specialists. Although surveys are a useful tool for gathering data from populations, they are inherently subject to bias and may not provide as detailed or objective information as the clinical assessment. This method limits our ability to assess speech and language development with the same precision as standardized evaluations.

Second, the development and validation process of the questionnaire is associated with some limitations. Although the items were carefully selected based on the relevant literature and expert input, we did not conduct formal reliability or validity testing of the survey (e.g., inter-reliability checks or internal consistency analyses). This could affect the consistency and robustness of the results, especially in terms of detecting subtle differences in speech development and speech disfluencies. Additionally, our survey assessed only several aspects of the descriptive information of the sample. The lack of information on participants’ gender and IQ, as well as parents’ age, ethnicity, educational level, etc., makes it impossible to analyze the impact of these variables on the results and limits the generalizability of our findings. Our experience gathered during this study indicates a need to expand the questionnaire and obtain a broader response. Another significant limitation is the exclusion of cognitive level variables and neuropsychiatric comorbidities in the analysis. Given that children with 22q11DS are at a higher risk of intellectual disabilities and conditions such as autism spectrum disorder (ASD), attention-deficit/hyperactivity disorder (ADHD), and anxiety disorders, the absence of these data limits our understanding of how these comorbidities might influence speech development. Additionally, while 22q11DS increases the risk of developing psychotic disorders such as schizophrenia, our study did not explore or discuss the potential connections between speech delay and the onset of psychosis. Although it was not within the scope of this study, this represents an important area for future research, which should incorporate cognitive assessment and neuropsychiatric evaluation to provide a more holistic view on the population.

Finally, the cross-sectional design of our study limits our ability to make conclusions about the long-term effects of early intervention on speech and language outcomes. Longitudinal studies are warranted to assess how early therapeutic interventions impact communication skills in children with 22q11DS. Further studies should also be performed on larger cohorts.

Nevertheless, our study was aimed at a preliminary assessment of the population of Polish children with 22q11DS due to the lack of studies on the Polish population in the context of speech and communication development, which is necessary for specialists to take a holistic approach in speech therapy. Despite these limitations, this study provides a foundation for future research on the speech and language profiles of children with 22q11DS in Poland, emphasizing the need for more comprehensive studies that incorporate clinical, cognitive, and neuropsychiatric evaluations.

## 5. Conclusions

This study sought to conduct an evaluation of the population of Polish children with 22q11DS. Our main motivation was the absence of a clinical description of this population regarding speech and communication development. We employed our own questionnaire that was approved by the 22q11 Poland Association and submitted to participants.

Based on the information from parents/guardians, we found that a considerable percentage of children presented with speech disfluencies, which suggests that it may be a common phenomenon associated with 22q11DS that has not been emphasized in previous studies. However, all observations should be confirmed using standardized tests for the Polish language. Further studies should be conducted to expand information on speech and communication in children with 22q11DS (their development, difficulties, and disorders that may impose limitations on them). Our findings are useful as regards the knowledge on 22q11DS, especially from the perspective of speech therapy. Discussing and systematizing them may contribute to increasing awareness and expanding the competence of specialists.

## Figures and Tables

**Table 1:** Time of babbling onset in the age groups (absent—did not occur, age given in years; months).

	Age of Babbling Onset					
Age Groups	Not Applicable	0;6–0;9	0;10–0;11	1;0 and over	Absent	Total
infants	1	1	0	0	0	2
1;0–1;11	0	2	2	0	1	5
2;0–2;11	0	2	0	2	2	6
3;0–6;11	0	4	7	6	2	19
7;0 and over	0	13	3	3	1	20
Total	1	22	12	11	6	52

**Table 2:** Time of the appearance of first words in the age groups (absent—did not occur, age given in years; months).

	Age of Using First Words						
Age Groups	Not Applicable	0;6–0;11	1;0–1;11	2;0–2;11	3;0 and over	Absent	Total
infants	2	0	0	0	0	0	2
1;0–1;11	0	1	3	0	0	1	5
2;0–2;11	0	1	4	0	0	2	7
3;0–6;11	0	1	9	5	5	0	20
7;0 and over	0	4	8	3	5	0	20
Total	2	7	24	8	10	3	54

**Table 3:** Time of the appearance of the first sentences in the age groups (absent—did not occur, age given in years; months).

	Age of First Utterances							
Age Groups	Not Applicable	1;0–1;11	2;0–2;11	3;0–3;11	4;0–4;11	5;0 and over	Absent	Total
infants	2	0	0	0	0	0	0	2
1;0–1;11	5	0	0	0	0	0	0	5
2;0–2;11	0	1	0	0	0	0	6	7
3;0–6;11	0	0	5	5	3	2	5	20
7;0 and over	0	5	4	2	2	7	0	20
Total	7	6	9	7	5	9	11	54

**Table 4:** Correlation results for the age of babbling onset, age of using first words, and age of using first sentences.

Kendall’s Tau Correlations			n	Kendall’s Tau B	*p*
Babbling (age)	-	First words (age)	44	0.368 **	0.001
Babbling (age)	-	First utterances (age)	33	0.357 **	0.007
First words (age)	-	First utterances (age)	35	0.593 ***	<0.001

** *p* < 0.01, *** *p* < 0.001.

**Table 5:** Participants’ age vs. preferred communication strategies (years; months age groups).

	Age Groups			
Prevalent Way of Communication	2;0–2;11	3;0–6;11	7;0 and Above	Total
gesture	3	3	0	6
individual words	3	4	0	7
utterances	0	7	0	7
longer statements	1	6	20	27
Total	7	20	20	47

**Table 6:** Results of the Mann–Whitney–Wilcoxon test: differences in the median age of acquiring milestones in children with and without disfluencies; no significant differences in age were found.

	U-Statistic	Median Age (in Months) in the Group Without Disfluencies	Median Age (in Months) in the Group with Disfluencies	*p*
Babbling (age)	201.0	9.0	11.5	0.291
First words (age)	228.0	18.0	24.0	0.481
First utterances (age)	113.5	36.0	48.0	0.263
Age	380.0	92.5	66.5	0.359

**Table 7:** The results of the Mann–Whitney–Wilcoxon test: differences in the median age of acquiring speech development milestones in children with and without hearing loss. No significant differences in age were found for babbling onset and using first sentences. The age when first words were used was significantly higher in children with hearing loss.

	U-Statistic	Median Age (in Months) in the Group Without Hearing Loss	Median Age (in Months) in the Group with Hearing Loss	*p*
Babbling (age)	140.5	9.0	11.0	0.140
First words (age)	121.5	18.0 *	33.0 *	0.011
First utterances (age)	78.0	41.0	54.0	0.087
Age	261.5	67.5	68.0	0.934

* *p* < 0.05.

**Table 8:** Hearing loss vs. orofacial dysfunction in the study population.

		Hearing Loss	
Orofacial Dysfunction	No	Yes	Total
no	16	4	20
yes	24	10	34
Total	40	14	54

**Table 9:** Age group vs. intelligibility of speech (age given in years; months).

	Who Understands the Child’s Speech?			
Age Groups	Only One Person	Some People	Everyone	Total
2;0–2;11	1	3	3	7
3;0–6;11	2	5	13	20
7;0 and over	1	9	10	20
Total	4	17	26	47

**Table 10:** Difficulties in interpersonal contact vs. age group (age given as years; months).

	Difficulties in Contact with Other People			
Age Groups	Never	Rarely	Always	Total
infants	2	0	0	2
1;0–1;11	4	1	0	5
2;0–2;11	3	1	3	7
3;0–6;11	7	5	8	20
7;0 and over	5	4	11	20
Total	21	11	22	54

**Table 11:** Kendall’s correlation results: age of the participants vs. frequency of difficulties in interpersonal contact.

Kendall’s Tau Correlations			Kendall’s Tau B	*p*
Age	**-**	Difficulties in contact with other people	0.317 **	0.003

** *p* < 0.01.

**Table 12:** The number of participants in speech therapy in the age groups (years; months).

	Did the Child Attend Speech Therapy?		
Age Groups	No	Yes	Total
infants	0	2	2
1;0–1;11	2	3	5
2;0–2;11	1	6	7
3;0–6;11	0	20	20
7;0 and over	0	20	20
Total	3	51	54

**Table 13:** Kendall’s correlation results: age of the participants vs. duration of speech therapy (months).

Kendall’s Tau Correlations			Kendall’s Tau B	*p*
Age	-	Duration of speech therapy	0.540 ***	<0.001

*** *p* < 0.001.

## Data Availability

The data presented in this study are available on request from the corresponding author (data are not publicly available).

## Appendix A

Survey on speech and communication development in children with the 22q11.2 deletion syndrome

*Original title**:* *Badanie rozwoju mowy i komunikacji dzieci z zespołem delecji 22q11.2*

******* *The original survey was constructed in Polish, as it was designed for the Polish-speaking community. Some questions included prompts based on Polish words and phrases typical of subsequent steps of speech development; the translation of the survey is given below.*

1. What age is your child? (Please provide the age in years and months, e.g., 0y 11m.; 7y 4m.)

*[Short open question]*

2. When was the microdeletion 22q11.2 syndrome diagnosed in your child?

*[Short open question]*

3. When did the child start babbling (syllables, e.g., *ma*, *pa*, *ta*, *da*; imitation of surrounding sounds)? If the question does not apply to your child, please write *not applicable*.

*[Short open question]*

4. What is the main mode of communication of the child ? *[single choice question]*
  ❑ crying
  ❑ screaming
  ❑ gestures
  ❑ single sounds
  ❑ single words
  ❑ single sentences
  ❑ longer statements (several sentences)
  ❑ other ……………………………………………………………………………….
5. When did the child start saying their first words? If the question does not apply to your child, please write *not applicable*.

*[Short open question]*

6. What were the first words? (please provide 3, writing them in the way the child pronounced them). If the question does not apply to your child, please write *not applicable*.

*[Short open question]*

7. When did the child start saying their first sentences (e.g., *Give me*!, *Come here*!)? If the question does not apply to your child, please write *not applicable*.

*[Short open question]*

8. Does the child diversify their signals (e.g., expresses hunger differently from boredom)? *[single choice question]*
  ○ always
  ○ often
  ○ rarely
  ○ not applicable
9. Are there any disfluencies in the speech of the child? *[single choice question]*
  ○ yes
  ○ no
10. If there are any disfluencies, what is their nature? *[multiple choice question]*
  ❑ blocks (difficulty in starting or finishing a word, e.g., m...ama)
  ❑ repetitions (of sounds, syllables, words, sentence fragments or whole sentences, e.g., m-m-m-ama; the car is, car is, car is red)
  ❑ prolongation (e.g., maaaaama)
11. Has the child been diagnosed with hearing loss? *[single choice question]*
  ○ yes
  ○ no
12. If yes, what kind of hearing loss is it? *[single choice question]*
  ○ conductive
  ○ receptive
  ○ mixed
  ○ I don’t know
13. Has the child ever been diagnosed with any orofacial dysfunction (i.e., lip/palate cleft disorders)? *[single choice question]*
  ○ yes
  ○ no
14. If yes, what type of disfunction was it? *[multiple choice question]*
  ❑ velopharyngeal insufficiency
  ❑ cleft lip
  ❑ hard palate cleft
  ❑ soft palate cleft
  ❑ submucous cleft palate
  ❑ cleft uvula
  ❑ I don’t know
  ❑ other ……………………………………………………………………………….
15. If yes, has the child undergone surgery to treat the dysfunction? *[single choice question]*
  ○ yes
  ○ no
16. If yes, what surgical procedure was performed and when ?

*[Short open question]*

17. Have you been informed about muscular tension disorder of the child’s articulation apparatus (i.e., lips, tongue, palate, facial muscles, etc.)? *[single choice question]*
  ○ yes
  ○ no
18. If yes, who provided such information?

*[Short open question]*

19. Who understands the speech of the child? *[single choice question]*
  ○ everyone
  ○ several people
  ○ only one person (e.g., the mother)
20. What types of communications are addressed to the child? *[multiple choice question]*
  ❑ simple words
  ❑ short sentences
  ❑ statements
  ❑ gestures
  ❑ words emphasized with gestures
  ❑ sentences emphasized with gestures
  ❑ other ……………………………………………………………………………….
21. Does the child understand the messages addressed to them? *[single choice question]*
  ○ always
  ○ frequently
  ○ rarely
  ○ never
22. Does the child understand the messages from anyone or only some individuals (who)?

*[Open question]*

23. Does the child show difficulties in interpersonal contact (e.g., starting conversation, eye contact, maintaining joint attention)? *[single choice question]*
  ○ yes
  ○ no
24. If yes, how often do such difficulties occur ? *[single choice question]*
  ○ always
  ○ frequently
  ○ rarely
25. If yes, in what circumstances do these difficulties occur? What might be the causes?

*[Open question]*

26. Has the child attended speech therapy? *[single choice question]*
  ○ yes
  ○ no
27. If yes, when did speech therapy start?

*[Short open question]*

28. If yes, how long has the child attended speech therapy?

*[Short open question]*

29. If yes, what was the reason for starting speech therapy? *[multiple choice question]*
  ❑ hearing loss
  ❑ articulation disorder
  ❑ speech and language delay
  ❑ speech disfluency
  ❑ cleft
  ❑ difficulties in feeding/eating
  ❑ poor/impaired communication with the environment
  ❑ other ……………………………………………………………………………….
30. If yes, in your opinion, which skills have been acquired/corrected due to speech therapy? What was the speech like before the therapy and what is it now?

*[Open question]*

31. Do you agree that speech therapists have sufficient knowledge on 22 chromosome deletion?
  ○ definitely agree
  ○ agree
  ○ disagree
  ○ strongly disagree

32. If you disagree/strongly disagree, what might be the cause?

*[Open question]*

If you would like to share any additional information related to the child’s speech and communication (e.g., their development, current state), there is some space below.
